# Evaluation of a Combinatorial Immunotherapy Regimen That Can Cure Mice Bearing MYCN-Driven High-Risk Neuroblastoma That Resists Current Clinical Therapy

**DOI:** 10.3390/jcm13092561

**Published:** 2024-04-26

**Authors:** Lauren Zebertavage, Allison Schopf, Megan Nielsen, Joel Matthews, Amy K. Erbe, Taylor J. Aiken, Sydney Katz, Claire Sun, Cole M. Witt, Alexander L. Rakhmilevich, Paul M. Sondel

**Affiliations:** 1 Department of Human Oncology, University of Wisconsin, Madison, WI 53705, USAaschopf@wisc.edu (A.S.); manielsen4@wisc.edu (M.N.); jamatthews3@wisc.edu (J.M.); aerbe@wisc.edu (A.K.E.); sakatz4@wisc.edu (S.K.); csun85@wisc.edu (C.S.); cmwitt2@wisc.edu (C.M.W.); rakhmil@humonc.wisc.edu (A.L.R.); 2 Department of Surgery, University of Wisconsin, Madison, WI 53705, USA; tjaiken@wisc.edu; 3 Department of Pediatrics, University of Wisconsin, Madison, WI 53705, USA

**Keywords:** neuroblastoma, MYCN, relapse, refractory, immunotherapy, GD2, immunocytokine, anti-CTLA-4, anti-CD40, CpG

## Abstract

**Background**: Incorporating GD2-targeting monoclonal antibody into post-consolidation maintenance therapy has improved survival for children with high-risk neuroblastoma. However, ~50% of patients do not respond to, or relapse following, initial treatment. Here, we evaluated additional anti-GD2-based immunotherapy to better treat high-risk neuroblastoma in mice to develop a regimen for patients with therapy-resistant neuroblastoma. **Methods**: We determined the components of a combined regimen needed to cure mice of established MYCN-amplified, GD2-expressing, murine 9464D-GD2 neuroblastomas. **Results**: First, we demonstrate that 9464D-GD2 is nonresponsive to a preferred salvage regimen: anti-GD2 with temozolomide and irinotecan. Second, we have previously shown that adding agonist anti-CD40 mAb and CpG to a regimen of radiotherapy, anti-GD2/IL2 immunocytokine and anti-CTLA-4, cured a substantial fraction of mice bearing small 9464D-GD2 tumors; here, we further characterize this regimen by showing that radiotherapy and hu14.18-IL2 are necessary components, while anti-CTLA-4, anti-CD40, or CpG can individually be removed, and CpG and anti-CTLA-4 can be removed together, while maintaining efficacy. **Conclusions**: We have developed and characterized a regimen that can cure mice of a high-risk neuroblastoma that is refractory to the current clinical regimen for relapsed/refractory disease. Ongoing preclinical work is directed towards ways to potentially translate these findings to a regimen appropriate for clinical testing.

## 1. Introduction

Neuroblastoma (NBL), a cancer derived from neural crest precursor cells, is the most common extra-cranial solid tumor in children. Patients diagnosed with NBL are segmented into prognostic categories with ~50% categorized as high-risk (HR). Recent studies have shown the benefit of adding anti-GD2 immunotherapy to the post-consolidation maintenance therapy for these patients, with clear improvement of both event-free survival and overall survival [1]. Of HR-NBL patients, ~40% are refractory to, or relapse following, initial treatment [2,3,4]. For HR-NBL patients that are refractory to, or relapse during or after, their initial therapy, recent testing has shown that the addition of anti-GD2 mAb to chemotherapy (most often temozolomide with irinotecan, but other regimens have also been effective) enables objective responses in ~40% of patients, a result far superior to chemotherapy alone [5,6]. While this is clear progress, fewer than 20% of these patients seem to remain disease free long-term [5]. The progress made in the use of haplo-identical stem-cell transplants with anti-GD2 mAb [7], in recent modifications of anti-GD2 CAR-T cells [8], and in the use of anti-GD2/anti-CD3 bispecific monoclonal antibody “armed” T-cells [9] suggests possible new approaches may be helpful for some of these patients that do not respond to “salvage” therapy. Even so, there are currently no widely-accepted, effective treatment options for these patients once they have failed salvage therapy of chemotherapy and anti-GD2 monoclonal antibody therapy [10].

In order to develop a regimen that may be effective in treating patients with neuroblastoma we have pursued anti-GD2-mAb-based combination therapies in immunocompetent mice bearing syngeneic, “cold” GD2-expressing tumors. We first showed that established B78 GD2-expressing melanomas were not responsive to immune checkpoint inhibition, but showed potent curative responses, with tumor-specific T-cell memory, when treated with a subtherapeutic dose (12 Gy) of local radiation therapy (RT) added to intratumoral anti-GD2 immunocytokine (a fusion protein of anti GD2 mAb and IL2) and anti-CTLA-4 [11,12]. We then asked if this same regimen was effective in treating 9464D-GD2, a murine NBL cell line from TH-MYCN transgenic mice, that we had transfected to provide stable membrane expression of GD2 [13]. We were surprised and disappointed that RT + anti-GD2 immunocytokine (IC) + anti-CTLA-4 did not slow the progressive growth of this tumor any more than the minimal slowing seen with 12 Gy RT alone. Several other immunotherapy (and other novel) regimens have been used on mice bearing this “cold” 9464D-GD2 neuroblastoma, and for the most, part tumor growth could be slowed in vivo, but rarely could mice be cured of measurable 9464D-GD2 tumors due to a variety of proposed mechanisms of resistance [14,15,16,17,18,19,20,21,22,23]. This refractory behavior was consistent with the low tumor mutation burden of this NBL (1/10 that of the B78 melanoma) [13], and consistent with the observation of potentially inhibitory macrophages and myeloid elements in this (and other) NBLs [24]. Our group previously published that the combination of an agonist anti-CD40 mAb together with the TLR9 activator, CpG, with or without chemotherapy could activate myeloid elements and replace M2 macrophages with M1 macrophages to induce partial anti-tumor effects in tumor bearing mice, including 9464D [25]. We thus added anti-CD40 mAb + CpG to the regimen of RT + intratumoral IC + anti-CTLA-4, and found that this regimen could now cure ~40–50% of mice bearing established, measurable tumors of this syngeneic 9464D-GD2 NBL [13,26]. We refer to this multi-agent package as our combination adaptive and innate immunotherapy regimen, “CAIR”. 

In order to consider the potential translation of this CAIR regimen to clinical testing, we wanted to **(1)** compare its efficacy with the current standard of care salvage chemo-immunotherapy regimen of temozolomide + irinotecan + anti-GD2 mAb [5], and **(2)** determine which of the five individual components of CAIR were essential for the anti-tumor efficacy we observe [13,26]. This report presents the results of our preclinical studies addressing these two issues. 

## 2. Methods

### 2.1. Cells

The parental 9464D cell line is a MYCN-driven neuroblastoma cell line derived from TH-MYCN transgenic mice [27]. 9464D-GD2 is a GD2-expressing cell line derived from 9464D as previously described [13], of which a clone was developed that does not express MHC-I even with IFNγ treatment [26]. Cells were grown in DMEM supplemented with 10% fetal bovine serum, 2 mM L-glutamine, 1 mM sodium pyruvate, 1X MEM non-essential amino acids, 100 U/mL penicillin, and 100 mcg/mL streptomycin. Cell lines were confirmed to be negative for mycoplasma by PCR prior to use. 

### 2.2. Murine Tumor Models

All mice procedures were conducted in accordance with the Institutional Animal Care and Use Committee at the University of Wisconsin–Madison. C57BL/6 female mice aged 6 to 8 weeks were purchased from Taconic Biosciences (Germantown, NY, USA). 9464D-GD2 flank tumors were engrafted by intradermal flank injection of 2 × 10^6^ tumor cells diluted in 100 μL phosphate-buffed saline (PBS). Dual-flank models required 2 × 10^6^ tumor cells to be engrafted by intradermal flank injection on both the right and left flank. Tumor size was determined by precision caliper measurement, and tumor volume was approximated using the formula (tumor volume in mm^3^) = [(tumor width in mm)^2^ × (tumor length in mm)]/2. Mice were randomized into treatment groups when tumors reached enrollment size (40–50 mm^3^ for single flank models and 1–5 mm^3^ for dual-flank models). The first day of treatment with RT was defined as “day 1”. After injection, approximately 90% of mice had tumors at the time of randomization that were suitably uniform to enable similar tumor sizes amongst all randomized mice (i.e., within the tumor volume range of 40–60 mm^3^ or 1–5 mm^3^); the remaining 10% of mice were excluded from randomization.

### 2.3. Radiotherapy

RT was delivered to primary tumors on day 1 of treatment using an Xstrahl Small Animal Radiation Research Platform (Suwanee, GA, USA). Mice were immobilized using custom lead jigs that exposed the dorsal right flank as previously described [26]. For all experiments, a maximum dose of 12 Gy RT was delivered to the right flank tumor in one fraction. In some settings, this RT approach was associated with local tumor ulceration. We found that this could be avoided by changing the radiation delivery from direct overhead with lead jig shields, to instead use a more restricted delivery to a smaller area/volume, at a 35-degree angle, under anesthesia using a SAARP collimator system (Xstrahl, Montreal, QC, Canada). 

### 2.4. Antibodies and Immunocytokine

Hu14.18-IL2 IC was provided by Anyxis (Vienna, Austria) and has been previously described [11,12]. The CAIR regimen, has been previously described [26] and includes the following agents. Intratumoral (IT) injections of 25 μg hu14.18-IL2 IC in 100 μL PBS were delivered once daily for 5 days (days 6 to 10). Anti-mouse-CTLA-4 mAb (αCTLA-4) (IgG2c isotype of the 9D9 clone) was provided by Bristol-Myers Squibb (Redwood City, CA, USA) and was administered intraperitoneally (IP) at a dose of 200 μg in 0.2 mL PBS on days 6, 9, and 12 as previously described. Anti-mouse-CD40 mAb (αCD40) was obtained from the ascites of nude mice injected with FGK 45.5 hybridoma cells producing agonistic anti-CD40 antibody (gift from Fritz Melchers, PhD, Basel Institute for Immunology, Basel, Switzerland). After enrichment for IgG, anti-CD40 mAb was administered IP at a dose of 500 μg in 0.2 mL PBS on day 3. CpG-1826 oligodeoxynucleotide (TCCTATGACGTCCCTGACGTT) was purchased from TriLink Biotechnologies (San Diego, CA, USA) or Integrated DNA Technologies (Coralville, IA, USA) and administered IT at a dose of 50 μg in 0.1 mL PBS on days 6, 8, and 10. Treatment timing was selected based on previous studies [26]. 

### 2.5. Chemotherapy

Temzolomide (TEM) was provided by Merck & Co (Rahway, NJ, USA) and Irinotecan (IRI) was provided by Pfizer Medical (New York, NY, USA); both have been previously described [28]. As described, TEM and IRI were previously administered once daily for 5 days (days 1 to 5) at 3.2 mg/kg and 1.6 mg/kg, respectively. For procedures used in this manuscript, mice were weighed prior to treatment each day and intraperitoneally (IP) injected with 3.2 μg TEM in 1 μL PBS and 1.6 μg IRI in 1 μL PBS per gram of mouse. The dosing used is comparable to previous experiments performed, and the treatment schedule was selected based on those studies [28].

### 2.6. Statistical Analyses

*p* values included in Tables were obtained by Log-rank [Mantel–Cox] test corresponding to survival plots shown in Figures. Statistical comparisons of tumor growth rate were performed by simple linear regression followed by one-way ANOVA analysis of the slopes of each curve with Tukey’s post hoc test for statistical considerations. Statistical comparisons of response rate (evaluating mice that became and remained tumor-free) were performed by Fisher’s Exact test.

## 3. Results

### 3.1. 9464D-GD2 Does Not Respond to the Combination of Temozolomide + Irinotecan + Anti-GD2

**Figure 1:**
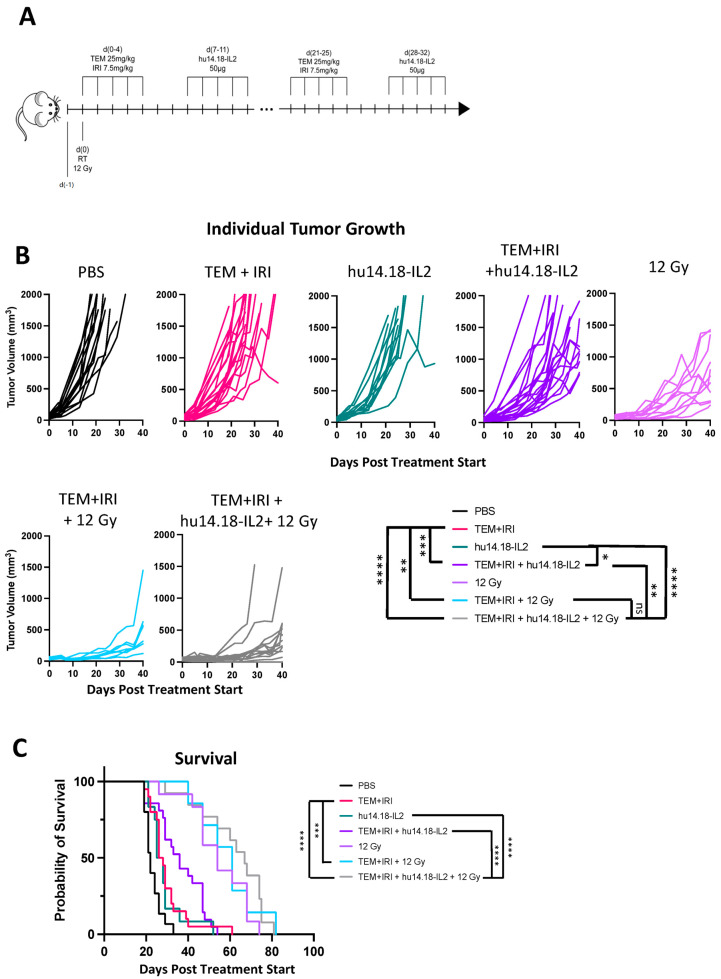
9464D-GD2 is not cured by TEM+IRI + anti-GD2 therapy (+/− RT). Cumulative results of 3 experiments testing radio-/chemo-/immunotherapy combinations in 9464D-GD2 tumors. (**A**) Treatment schedule for mice receiving TEM+IRI, hu14.18-IL2, and/or 12Gy external beam radiotherapy. (**B**) Individual tumor growth curves are shown for each treatment group, as indicated. Select statistical comparisons are shown, with complete statistical results found in Supplemental Table S1. (**C**) Kaplan–Meier survival plot and table of corresponding *p*-values (Log-rank [Mantel–Cox] test), with select statistical comparisons shown. ns = not significant; * *p* < 0.05; ** *p* < 0.01; *** *p* < 0.001; **** *p* < 0.0001.

While established 40–50 mm^3^ 9464D-GD2 tumors virtually all respond to CAIR with initial tumor shrinkage, and ~40–50% are cured by this regimen [13,26], we wanted to see how this compares to treatment of comparable tumors with the clinical standard of care, namely TEM+IRI + anti-GD2 mAb. C57BL/6 mice bearing 40–50 mm^3^ 9464D-GD2 tumors, that had been growing for ~4 weeks to reach this size, were thus treated. Our prior work had shown that intratumoral (IT) treatment delivered more anti-GD2 agent to the tumor than IV treatment [29], and that IT hu14.18-IL2 immunocytokine (IC) induced a more potent immunotherapeutic effect than the same dose of IV hu14.18-IL2. Thus, to test TEM+IRI with anti-GD2 mAb, we chose to test it with the more potent IC (instead of the anti-GD2 mAb), given by IT delivery (Figure 1A). Figure 1B shows that temazolomide with irinotecan (without IT-IC), and IT-IC alone, show minimal slowing of the progressive tumor growth compared to PBS. While the chemotherapy in combination with anti-GD2 also shows some slight slowing of the tumor growth, the tumors continue to grow progressively (Figure 1B). Most importantly, with respect to clinical translation, all mice in these four groups are all dead by day 62 (Figure 1C). In the PBS group, all mice are dead by day 33. While survival is slightly prolonged for the chemo alone (*p* < 0.01) and IT-IC alone (*p* = 0.03) groups (*p* values for survival for all possible comparisons shown in Figure 1C, are included in Table 1), all mice in these three groups [PBS, TEM+IRI, IC] die by day 55 (with one outlier surviving in the chemotherapy alone group to day 62). Over >90% of the mice in these three groups are dead by day 40. When TEM and IRI are combined with IT-IC, all mice are dead by day 55; survival is not significantly increased compared to chemotherapy alone (*p* = 0.054) or to IT-IC alone (*p* = 0.060). This lethality from progressive tumor for all these mice receiving chemotherapy plus IT-IC is different from the ~40–50% long-term tumor-free survival we have seen with the use of IT-IC in CAIR [13,26] for comparable sized tumors. 

As established measurable GD2^+^ B78 tumors do not respond to IT-IC, or to RT, but are cured when RT is combined with IT-IC [12,13], we wondered if adding 12 Gy RT (as used in the CAIR regimen) to TEM+IRI + IT-IC would show meaningful anti-tumor activity for 9464D-GD2. Figure 1 shows that this is not the case. Radiation alone slows tumor growth (Figure 1B) and significantly prolongs survival (Figure 1C), compared to PBS (*p* < 0.001). However, all RT-treated mice are still dying of progressive tumor before day 80. Adding TEM+IRI, with or without IT-IC to the RT, does not significantly prolong survival (*p* = 0.57, for TEM+IRI without IC, and *p* = 0.10 for TEM+IRI with IC), and all animals in these two groups are dead by day 82. Statistical data for comparisons between all treatment groups for tumor growth (Figure 1B) are included in Supplemental Table S1. These data show that combining IT-IC with TEM+IRI does not meaningfully impact survival of 9464D-GD2-bearing mice, either with or without RT. Moreover, these studies in Figure 1 show that in mice, the 9464D-GD2 behaves similarly to the cancers in ~60% of patients with relapsed/refractory HR-NBL. Namely, they are not responding to this chemo-immunotherapy approach.

### 3.2. The Radiotherapy and the IT-IC Components Are Each Essential to Maintaining the Anti-Tumor Potency of CAIR

The CAIR regimen cures ~50% of C57BL/6 mice bearing ~4 week established ~50 mm^3^ 9464D-GD2 NBL tumors [13,26]. In the process of developing this regimen, we had previously shown that adding IT-IC and anti-CTLA-4 mAb to 12 Gy of RT did not significantly slow tumor growth, or prolong survival, over the 12 Gy of RT alone. We hypothesized that adding activation of innate immune cells with anti-CD40 + CpG to the RT + IC + anti-CTLA-4 combination would provide an additional pathway to enable greater anti-tumor efficacy. As curative responses were seen, this indicated a beneficial augmented anti-tumor effect; this combination thus became our CAIR regimen [13,26]. Our data demonstrated that virtually all mice bearing ~50 mm^3^ 9464D-GD2 tumors showed initial tumor shrinkage and ~50% remained disease-free long-term [13]. Since creating this preclinical regimen, we wanted to determine which components of it were essential for the beneficial anti-tumor effect. 

**Figure 2:**
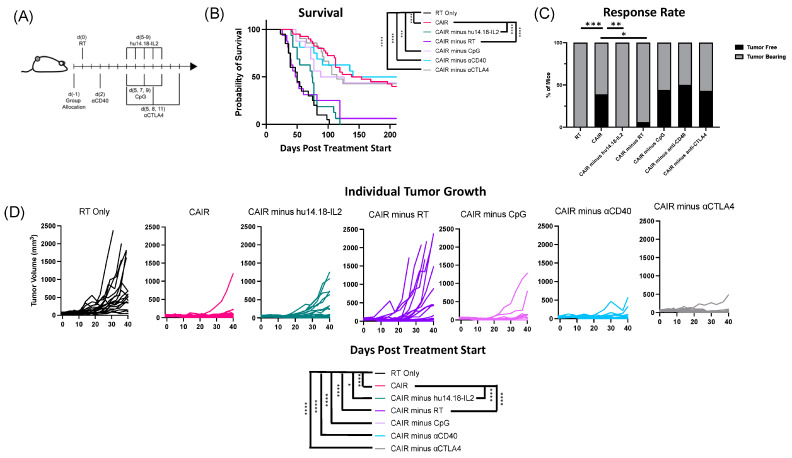
Radiotherapy and hu14.18-IL2 therapy are necessary for the curative response of 9464D-GD2 tumors to CAIR. (**A**) Treatment schematic for full CAIR regimen, (**B**) Kaplan–Meier survival plot for 9464D-GD2-bearing mice treated as indicated (RT Only = radiotherapy, 12Gy; CAIR = full regimen shown in (**A**); CAIR minus X = regimen shown in (**A**) without X component). (**C**) Response rates are shown, with the percentage of mice that became and remained tumor-free (black) graphed together with the percentage of mice that remained tumor-bearing (gray) for each treatment group. (**D**) Tumor growth curves for mice treated as indicated in (**A**,**B**). Data shown are compiled from 2–4 individual experiments, with n = 16–40 per group. Days Post Treatment Start = time after radiation or sham radiation administration. ns = not significant; * *p* < 0.05; ** *p* < 0.01; *** *p* < 0.001; **** *p* < 0.0001.

Here, in four separate experiments, we tested five separate CAIR regimen variants, where we individually removed one of the five components (RT, IC, anti-CTLA-4, anti-CD40, or CpG), and compared them to the full CAIR regimen or to RT alone (which has no efficacy against 9464D-GD2 tumors) (Figure 2). Figure 2A shows the treatment schema; Figure 2B shows the survival curves; and Figure 2C shows the number of mice that became and remained tumor-free in each of the seven groups, and the tumor growth curves for each of the individual mice in each of these seven separate treatment groups are shown in Figure 2D. Key statistical comparisons are shown within Figure 2B–D. Based on the survival curves in Figure 2B, removing either RT or IC from the CAIR regimen caused a complete abrogation of the immunotherapeutic effect; the survival curves of CAIR minus hu14.18-IL2 and CAIR minus RT are not significantly different from the survival curve of RT alone. In these three groups, nearly all mice died before day 120. Thus, both RT and hu14.18-IL2 are essential for the anti-tumor survival efficacy of CAIR. This is consistent with the results in Figure 2C,D; in both of these, the only three groups that are significantly different from CAIR are RT alone, CAIR minus RT, and CAIR minus hu14.18-IL2. In contrast, the survival, tumor cure rate, and tumor growth data for CAIR minus CpG, CAIR minus anti-CD40, and CAIR minus anti-CTLA-4 in Figure 2B–D are not significantly different from that of the full CAIR regimen. This indicates that, under these conditions, these three agents, individually, do not contribute to the observed anti-tumor efficacy of the full CAIR regimen. Table 2 provides a summary of the data presented in Figure 2C, clarifying the number of animals in each group, the number of cured mice, and the number of separate experiments providing data for Figure 2C. Statistical comparisons between all treatment groups for the survival data in Figure 2B are included in Table 3. Statistical data for comparisons between all treatment groups for tumor growth (Figure 2D) are included in Supplemental Table S2, and for the response rate in Supplemental Table S3.

### 3.3. The Potency of the CAIR Regimen Is Abrogated by Eliminating CD40 with Either CpG or Anti-CTLA-4

In contrast to the requirement for RT and IC in the efficacy of the CAIR regimen, the elimination of either CPG, anti-CD40, or anti-CTLA-4 alone, did not cause a either a visible change (or a statistically significant change) in the efficacy of the full CAIR regimen to prolong survival or cure mice (Figure 2B,C). Yet, our prior data indicated that substantial benefit occurred when anti-CD40 and CpG were added together to RT + IC + anti-CTLA-4 [13]. Thus, in this study, we asked whether removing two out of three of these agents (anti-CD40, CpG, and anti-CTLA-4) might allow us to retain the efficacy of the CAIR regimen, using only three of the five separate components.

**Figure 3:**
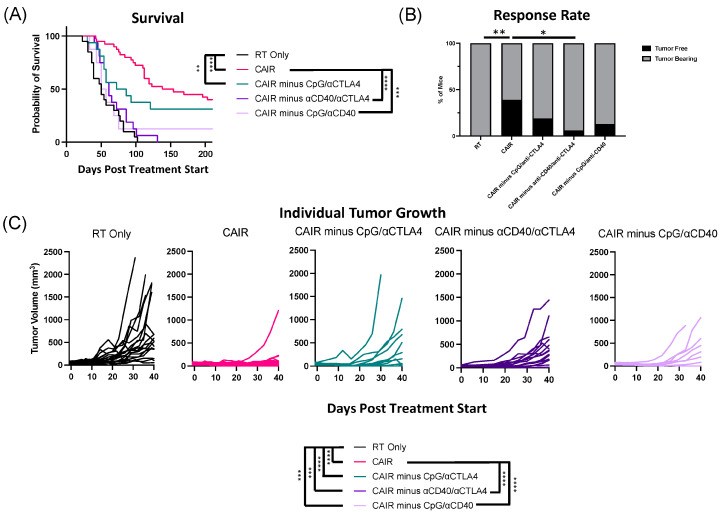
CpG, anti-CD40, and anti-CTLA4 are dispensable in isolation but cannot be removed in combination while maintaining curative efficacy of CAIR. (**A**) Kaplan–Meier survival plot for animals bearing 9464D-GD2 tumors treated as indicated (RT Only = radiotherapy, 12Gy; CAIR = full regimen shown in Figure 2A; CAIR minus CpG/anti-CTLA-4, CAIR minus anti-CD40/anti-CTLA-4, CAIR minus CpG/anti-CD40), (**B**) Response rates are shown, with the percentage of mice that became and remained tumor-free (black) graphed together with the percentage of mice that remained tumor bearing (gray) for each treatment group. (**C**) Tumor growth curves for mice treated as indicated in (**A**,**B**). Days Post Treatment Start = time after radiation administration. Data shown are compiled from 1–4 individual experiments, with n = 8–40 per group. ns = not significant; * *p* < 0.05; ** *p* < 0.01; *** *p* < 0.001; **** *p* < 0.0001.

In the same four experiments evaluated together in Figure 2, we also tested the CAIR regimen minus: (1) CpG and anti-CTLA-4; (2) anti-CD40 and anti-CTLA-4; and (3) CpG and anti-CD40 (Figure 3). For comparison, we include in Figure 3 the control groups of RT alone and the full CAIR regimen (as also shown in Figure 2). The schema for this is shown in Figure 2A, the survival curves are shown in Figure 3A, the tumor response rate is shown in Figure 3B and the tumor growth curves for all animals in each of the 5 separate treatment groups are shown in Figure 3C. The survival curves in Figure 3A show that eliminating the combination of anti-CD40 and anti-CTLA-4, or eliminating the combination of CpG and anti-CD40, each bring the survival down dramatically and significantly compared to the full CAIR regimen; neither curve is significantly better than the RT alone group. In contrast, the elimination of the CpG and anti-CTLA-4 combination appears to bring the survival down somewhat from the full CAIR regimen, but this difference is not significant, while the survival for this group remains significantly better than that of the RT alone group. Although the CAIR group survival is not significantly better than that for the CAIR minus CpG + anti-CTLA-4 group, the *p* value is close to a “trend” (*p* = 0.1254), and the position of the survival curve for this latter group looks intermediate between CAIR and RT (Figure 3A), as if more extensive testing (namely more experiments with a greater number of animals per group) may well have the statistical power to show that the CAIR minus CpG + anti-CTLA-4 combination might actually deliver survival that is better than RT alone, but also worse than the full CAIR regimen. 

The tumor growth curve data in Figure 3C is consistent with the survival results in Figure 3A. Namely, the CAIR minus CpG/anti-CTLA-4 group is not significantly worse than the full CAIR regimen, while both the CAIR minus anti-CD40/anti-CTLA-4 group and the CAIR minus CpG/anti-CD40 group are significantly worse than the full CAIR regimen. The tumor response rate in Figure 3B also shows that CAIR is significantly superior to CAIR minus anti-CD40/anti-CTLA-4, but there is not sufficient statistical power to determine whether it is superior to CAIR minus CPG/anti-CTLA-4 or CAIR minus CpG anti-CD40. The data for the tumor response in Figure 3B are also presented in Table 2 (along with the tumor response data from Figure 2C). The statistical comparisons for survival shown in Figure 3A are all presented in Table 3, along with the comparisons for the survival data shown in Figure 2B. Statistical data for comparisons between all treatment groups for tumor growth shown in Figure 3C (together with the comparisons for tumor growth in Figure 2D) are included in Supplemental Table S2. Statistical data for comparing the response rate of all groups (as shown in Figure 2C and Figure 3B) to each other are included in Supplemental Table S3. 

### 3.4. Efficacy of the CAIR Regimen on a Much Smaller Distant Tumor

Our initial description of the CAIR regimen demonstrated ~50% of mice treated with ~50 mm^3^ 9464D-GD2 tumors became and stayed tumor-free, as shown here in Figure 2 and Figure 3, and noted that larger tumors (~100 mm^3^ or larger) might be slightly slowed in their growth by CAIR but would not become or remain tumor-free [13,26]. We thus wanted to confirm that the CAIR regimen would still be effective for substantially smaller tumors. We chose here to test the CAIR minus anti-CD40 regimen for 2 reasons. First, as Figure 2 shows this regimen is as effective as CAIR for 40–50 mm^3^ tumors, we thought it would also be effective against smaller tumors. Secondly, clinical and preclinical use of anti-CD40 has been associated with some systemic dose-dependent toxicity, likely reflecting myeloid cell activation resulting in cytokine release and necroinflammatory liver disease potentially influenced by the microbiome [30,31,32,33]. We have also seen dose-limiting systemic toxicity in C57BL/6 mice bearing 9464D-GD2 tumors treated with single-agent anti-CD40, in the form of transient weight loss (~10% of body weight) lasting 2–4 days following systemic administration (Supplemental Figure S1). 

**Figure 4:**
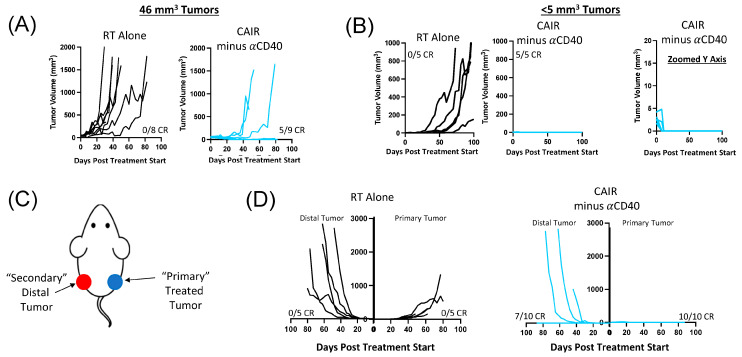
CAIR minus anti-CD40 efficacy is dependent on 9464D-GD2 tumor size and is associated with systemic cures of very small tumors. (**A**) Tumor growth curves of 9464D-GD2 tumors that were treated at ~46 mm^3^ with 12 Gy radiotherapy (RT Only) or with radiotherapy, hu14.18-IL2, CpG, and αCTLA4 (CAIR Minus αCD40). (**B**) Tumor growth curves of 9464D-GD2 tumors that were treated at <5 mm^3^ with 12 Gy RT or with CAIR Minus αCD40. The middle panel and the panel on the right show the exact same data, with the panel on the R showing a much more limited tumor size scale on the Y-axis, to enable visualization of all 5 of these tumors growing initially, and then shrinking by day 16. (**C**) Mouse diagram denoting location of primary (directly treated) and secondary (distal) flank tumors for the data in (**D**). (**D**) Tumor growth curves of mice with 2 similar <5 mm^3^ 9464D-GD2 tumors, one in the L and one in the R flank; (**D-left graph**) the 2 panels show the tumor on the L flank and R flank, marked as distal and primary tumor, respectively, in mice treated with RT to the R flank only; (**D-right graph**) the 2 panels show the tumor on the L flank (distal) and R flank (primary) as marked, in mice receiving RT to the R flank, IT IC and IT-CpG to the R tumor only, and systemic anti-CTLA-4 (CAIR minus anti-CD40); comparable findings to these were obtained in a similar experiment. For 8 of these 9 graphs, above the *x*-axis in each is a designation “X/Y CR” where Y is the number of mice with measurable tumors at that site that received treatment, and X is the number of mice that became and remained tumor-free at that site as of day 87 after treatment initiation.

For this study, mice were implanted with 9464D-GD2 tumors at different times, so that the tumors implanted for ~4 weeks had a mean volume of 46 mm^3^ (17 mice). A separate group of mice implanted for only ~2 weeks showed some mice without detectable tumors, but 10 mice had clearly palpable tumors, where rough measurements could be estimated (<5 mm^3^). The mice with the larger tumors were randomized into two groups, one receiving RT alone and the other receiving CAIR minus anti-CD40. The mice bearing smaller tumors were also randomized to either RT or CAIR minus anti-CD40. The growth curves for the 17 mice with the larger tumors in Figure 4A shows progressive growth for all eight of the tumors receiving RT alone, with five out of nine mice receiving CAIR minus anti-CD40 becoming and remaining tumor-free, consistent with the data shown in Figure 2B–D. Comparable data are shown for the 10 mice with the smaller tumors in Figure 4B The left panel shows that even for these very small tumors, the RT does not have a curative effect for any mice; five out of five mice show progressive tumors. In contrast, in the middle panel, five of five mice receiving the CAIR-minus anti-CD40 treatment became and remained tumor-free, consistent with our prior reports of greater immunotherapeutic efficacy for smaller tumors [34]. The far right panel in Figure 4B shows the same data as the middle panel, but the Y axis is at a different scale to show that all five of these tumors actually grew slightly up to day 8 after starting treatment, but then resolved completely by day 16.

As these smaller tumors in Figure 4B showed a more uniform response to the CAIR minus anti-CD40 regimen, we asked whether the locally administered components of this regimen (the RT and the IT-CpG and IT-IC) needed to be delivered directly to the small tumor to have this potent anti-tumor effect. To test this, mice were implanted with 9464D-GD2 tumors at the same time in both the left and right flank (schema in Figure 4C). Approximately 2 weeks later, when the bilateral tumors were barely measurable, 15 mice with palpable bilateral tumors, all <5 mm^3^, were randomized at a 1:2 ratio, with 5 mice receiving RT alone only to the R flank tumor, while the remaining 10 mice received RT and IT-CpG and IT-IC only to the R flank tumor, together with systemic administration of anti-CTLA-4. Figure 4D shows the data for both L and R tumors in mice treated with RT alone to the R tumor. The RT-treated tumors (in the R flank) all grew progressively (consistent with data shown for treatment with RT alone in the L panel of Figure 4C), but slowly, with all tumors receiving RT still <250 mm^3^ as of day 44 (the day the first animal needed sacrifice due to rapid progression of the tumor on the L). In contrast, the non-irradiated tumors on the L flank, in these same mice that received RT to the R tumor, all grew progressively, but much more rapidly than the RT-treated tumor on the R; as of day 44, four of five tumors were >250 mm^3^. In contrast, the R panel of Figure 4D shows the data for both the L and R flank tumors in mice treated with CAIR minus anti-CD40, where the RT and IT injections (of CpG and IT-IC) were only given to the tumor on the R flank and the anti-CTLA-4 was given systemically. These mice demonstrate dramatic anti-tumor responses. The tumors on the R flank, receiving both local and systemic treatment, completely resolved, with this site remaining tumor-free for all 10 treated mice. In addition, even for the tumors on the L flank, when the RT, IT-IC, and IT-CpG were given to the contralateral tumor (with systemic anti-CTLA-4), 7 out of these 10 tumors resolved and did not recur. Separate preliminary work has repeated these findings and has also shown that these small tumors (as in the L flank tumors in the CAIR minus anti-CD40-treated mice in Figure 4D) do not respond to systemic anti-CTLA-4 treatment alone. This suggests that even though the CpG and IC were given intratumorally to the opposite tumor, a sufficient level of these agents spread systemically from the R tumor to reach the L tumor to have a clear immunotherapeutic effect (when combined with the systemic anti-CTLA-4). We have previously demonstrated that IC given intratumorally initially provides very high local concentrations at the IT injection site, but with time, the agent diffuses out and meaningful concentrations can reach a distant tumor [29].

## 4. Discussion

The incorporation of anti-GD2 mAb into the standard of care for patients with HR-NBL is clearly having an impact on improving response and survival, both in the treatment of newly diagnosed patients and in the treatment of relapsed/refractory patients [2,5,10]. Even though the addition of anti-GD2 mAb to chemotherapy is increasing response rates and survival for relapsed/refractory patients, nearly 90% either do not respond or end up relapsing [5,35]. We have focused here on the 9464D-GD2 NBL, which, like in many patients with high-risk disease, is driven by MYCN [13,24,36]. Many other murine tumors (including some NBLs) studied in preclinical models, have relatively high tumor mutation burdens, and can respond to regimens that can induce T cell responses [13,24,29,35], but these do not simulate the low tumor mutation burden of most clinical pediatric cancers. In contrast this 9464D-GD2 NBL has a relatively low tumor mutation burden of 1.5 mutations per megabase, and no MHC-I expression, providing potential explanations for why it does not respond to immunotherapy regimens that induce T-cell dependent, curative responses by more immunogenic tumors [13,26]. In this study, we show that this 9464D-GD2 NBL is also refractory to a somewhat standard regimen of chemotherapy + anti-GD2 mAb, which is frequently used clinically [5]. As anti-GD2 regimens can be effective in the absence of T cells, the mechanism of action is thought to involve antibody dependent cell-mediated cytotoxicity (ADCC) by NK cells and other immune cells bearing Fc receptors [37]. Further studies are needed to better characterize why this 9464D-GD2 NBL is refractory in vivo to regimens that likely work via ADCC, since 9464D-GD2 is highly susceptible to ADCC in vitro [13]. 

Even so, this refractoriness of 9464D-GD2 to the TEM+IRI + anti-GD2 regimen, shown here, suggests that this 9464D-GD2 model behaves similarly to the HR-NBL of the majority of patients with refractory or relapsed NBL who do not respond to, or relapse following, clinical treatment with this same regimen [2,5]. For this reason, we focused on developing a preclinical regimen that could provide meaningful in vivo anti-tumor responses against 9464D-GD2. Our goal has been to obtain in vivo complete responses, with sustained absence of recurrence. We previously published our work showing that the CAIR regimen could cure ~50% of mice bearing ~50 mm^3^ 9464D-GD2 flank tumors, and that this response did not appear to involve T cells [11,14]. We showed that there was initially a relative paucity of tumor-infiltrating T cells; however, following CAIR treatment, there was a significant increase in CD4 and CD8 T cells with a significant decrease in Treg cells [26]. This regimen included both anti-CD40 mAb and CpG, agents being used clinically, but also associated with some toxicity likely related to cytokine secretion [37,38]. Here, we asked whether we could obtain a similar efficacy using the backbone of the CAIR regimen while determining which components of it were essential. We found that the efficacy of the CAIR regimen was lost if either the RT or IC was eliminated, but the efficacy of the CAIR regimen was retained if any one of the anti-CTLA-4, anti-CD40, or CpG components were removed (Figure 2). Intriguingly, anti-tumor activity was lost when anti-CD40 was eliminated together with either anti-CTLA-4 or CpG, while some anti-tumor activity was retained when the anti-CTLA-4 and CpG were both eliminated (Figure 3). 

Furthermore, when testing this approach against much smaller 9464D-GD2 tumors (<5 mm^3^), RT alone had no curative effect, while the CAIR regimen was uniformly curative (Figure 4B), and even showed anti-tumor activity against distant very small tumors (Figure 4D). Although not proven, this suggests there may be efficacy of this combination against distant microscopic metastases, not measurable clinically.

Recently, a separate approach using local intratumoral injection of Prussian blue nanoparticles and CpG into small 9464D tumors has induced both complete responses and immune memory. These important results were obtained with a 9464D variant found to express MHC-I and MHC-II, which were further induced with this immunotherapy [39]. In contrast, the 9464D-GD2 tumor line we have used in this study does not express MHC-I, and cannot be induced to express MHC-I by interferon-γ treatment, making this tumor more similar to the refractory human NBLs that do not have inducible MHC [26]. 

We have previously noted that the addition of both CpG and anti-CD40, in combination, added transient toxicity to the RT + IT-IC in the form of lethargy and transient weight loss, likely reflecting increased cytokine release occurring due to the immunostimulatory co-interactions of the IL2 of the IC, CpG, and anti-CD40 [13]. In prior work [13], we also noted that this toxicity was improved by lowering (by 50%) the dose of the IT-IC or the anti-CD40, without a detectible decrease in anti-tumor efficacy. Even though we found here that anti-CD40 can, by itself, be associated with transient weight loss (Supplemental Figure S1), we anticipate that our future analyses to titrate the dose of anti-CD40 will enable a dose that can provide efficacy with minimal or tolerable toxicity. 

Recently, anti-CD40 agonist antibodies have been used in a variety of preclinical and clinical settings to induce augmented anti-tumor efficacy in a variety of regimens and combinations. Some of the preclinical work has been performed with NBL. In mice, agonist anti-CD40 mAb can augment the anti-tumor efficacy of an anti-GPC2 antibody drug conjugate via macrophage activation [40], and can also facilitate the induction of anti-survivin reactive T cells that kill slightly immunogenic NBLs, likely via antigen-presenting cell activation [41]. Some human NBLs express CD40, and in vitro treatment with a CD40 agonist can induce tumor cell apoptosis [42]. There is a growing body of clinical testing in more common cancers in adults. In these more immunogenic tumors, the focus has been more on the activation of antigen-presenting cells via anti-CD40 mAb to help activate tumor-reactive T cells [30,43,44,45,46,47].

Further preclinical work is underway to use these results in the development of improved combinations that might be well tolerated clinically, with a focus on treating neuroblastoma patients not responding to, or responding incompletely to, current chemo-immunotherapy regimens like temozolomide, irinotecan, and anti-GD2 mAb. While this approach is providing long-term benefit for mice with a single ~50 mm^3^ flank tumor, additional preclinical work is needed to generate a more effective and well-tolerated regimen that may be able to eradicate larger tumors, especially in the setting of metastatic disease, as is often seen clinically. 

## 5. Conclusions

Here, we demonstrate that 9464D-GD2 tumors behave similarly to human HR-NBLs that fail to be cured by salvage therapy. Relatively small flank tumors do not respond to temozolamide + irinotecan + anti-GD2 mAb (Figure 1) at concentrations somewhat comparable to those used clinically [28]. In contrast, these tumors can be cured (Figure 2 and Figure 3) by both CAIR therapy, and by several versions of a reduced CAIR therapy (CAIR without αCD40, αCTLA-4, or CpG, or CAIR without CpG/αCTLA-4). Ongoing preclinical work is directed towards ways to potentially translate these findings to a regimen appropriate for clinical testing.

## Figures and Tables

**Table 1:** *p* values for all comparisons of survival shown in Figure 1C. *p* values <0.05 are in **bold**.

	PBS	TEM+IRI	hu14.18-IL2	TEM+IRI + hu14.18-IL2	12 Gy	TEM+IRI + 12 Gy	TEM+IRI + hu14.18-IL2 + 12 Gy
PBS		**0.0046**	**0.0303**	**<0.0001**	**<0.0001**	**<0.0001**	**<0.0001**
TEM+IRI			0.5347	0.0536	**<0.0001**	**0.0002**	**<0.0001**
hu14.18-IL2				0.0603	**<0.0001**	**0.0002**	**<0.0001**
TEM+IRI + hu14.18-IL2					**0.0003**	**0.0006**	**<0.0001**
12 Gy						0.5663	0.1003
TEM+IRI + 12 Gy							0.8681
TEM+IRI + hu14.18-IL2 + 12 Gy							

**Table 2:** Cure rate for all groups shown in Figure 2C and Figure 3B. For each treatment group in the first column, the next column shows the number of independent experiments including that group. The 3rd column shows for each group, the number cured in that treatment group (tumor-free as of day 150)/the total number of tumor-bearing mice treated in that group, followed by the % cure rate.

Treatment	Independent Experiments	Cure Rates
CAIR	4	15/38 = 39%
CAIR minus hu14.18-IL2	2	0/16 = 0%
CAIR minus RT	2	1/16 = 6%
CAIR minus CpG	2	7/16 = 44%
CAIR minus αCD40	2	8/16 = 50%
CAIR minus αCTLA4	3	9/21 = 43%
CAIR minus CpG/αCTLA4	2	3/16 = 19%
CAIR minus αCD40/αCTLA4	2	1/16 = 6%
CAIR minus CpG/αCD40	1	1/8 = 13%
RT	3	0/19 = 0%

**Table 3:** *p* values, comparing the survival curves for each group shown in Figure 2B and Figure 3A to all other groups in Figure 2B and Figure 3A are provided. For consistency in comparison, the exact same CAIR group and RT group are included identically in Figure 2B and Figure 3A. *p* values < 0.05 are in **bold**.

	RT Only	CAIR	CAIR Minus CpG	CAIR Minus αCD40	CAIR Minus αCTLA4	CAIR Minus RT	CAIR Minus hu14.18-IL2	CAIR Minus CpG/αCTLA4	CAIR Minus αCD40/αCTLA4	CAIR Minus CpG/αCD40
RT Only		**<0.0001**	**0.0002**	**<0.0001**	**<0.0001**	0.2563	0.0621	**0.0046**	0.1730	0.5602
CAIR			0.7724	0.7204	0.8719	**<0.0001**	**<0.0001**	0.1254	**<0.0001**	**0.0003**
CAIR minus CpG				0.6140	0.7443	**0.0033**	**0.0036**	0.3285	**0.0017**	**0.0125**
CAIR minus αCD40					0.7684	**0.0007**	**0.0002**	0.1898	**0.0002**	**0.0070**
CAIR minus αCTLA4						**0.0008**	**<0.0001**	0.1939	**<0.0001**	**0.0020**
CAIR minus RT							0.9635	**0.0422**	0.8181	0.8105
CAIR minus hu14.18-IL2								0.0586	0.8580	0.6308
CAIR minus CpG/αCTLA4									0.0653	0.1639
CAIR minus αCD40/αCTLA4										0.8717
CAIR minus CpG/αCD40										

## Data Availability

Raw data comparing the tumor mutation burden of 9464D cell line variants were generated in our colleague John Maris’s lab, at the Children’s Hospital of Philadelphia. Derived data supporting the findings of this study are available from the corresponding author, PMS, upon request.

## Supplementary Materials

The following supporting information can be downloaded at: https://www.mdpi.com/article/10.3390/jcm13092561/s1, Figure S1: C56BL/6 mice bearing 17 mm^3^ 9464D-GD2 tumors were not treated, or received anti-CD40 (0.5 mg/mouse intraperitoneally) on days 1 and 8. Mouse weights were obtained every 2–3 days. There were 5 mice per group. Mouse weight dropped ~10% over 4 days following the first dose, with minimal weight loss after the 2nd dose; Table S1: *p* values, comparing the tumor growth curves for each group shown in Figure 1B; Table S2. *p* values, comparing the tumor growth curves for each group shown in Figure 2D and Figure 3C. For consistency in comparison, the exact same CAIR group and RT group are included in Figure 2 and Figure 3; Table S3. *p* values, comparing the response rate for each group shown in Figure 2C and Figure 3B. For consistency in comparison, the exact same CAIR group and RT group are included in Figure 2C and Figure 3B.
