# Supplementary material for: Evaluation of a Combinatorial Immunotherapy Regimen That Can Cure Mice Bearing MYCN-Driven High-Risk Neuroblastoma That Resists Current Clinical Therapy
Source: J Clin Med. 2024 Apr 26;13(9):2561. doi: 10.3390/jcm13092561 (PMC11084214; doi:10.3390/jcm13092561)
Supplement: Supplementary file 1 [file jcm-13-02561-s001.zip › jcm-2906048-supplementary.pdf]

Supplementary Table S1: Statistical output of comparisons of tumor growth for each treatment group represented in Figure 1

Treatment	PBS	TEM+IRI	IC	TEM+IRI + IC	12 Gy	TEM+IRI + 12 Gy
**TEM+IRI**	****					
**IC**	***	ns				
**TEM+IRI + IC**	****	ns	*			
**12 Gy**	****	***	****	*		
**TEM+IRI + 12 Gy**	****	**	****	ns	ns	
**TEM+IRI + IC + 12 Gy**	****	****	****	**	ns	ns

ns = not significant; *p<0.05; **p<0.01; ***p<0.001; ****p<0.0001

Supplementary Table S2: Statistical output of comparisons of tumor growth for each treatment group represented in Figures 2 and 3. p values <0.05 are highlighted.

Treatment	CAIR	CAIR minus CpG	CAIR minus αCD40	CAIR minus αCTLA4	CAIR minus RT	CAIR minus IC	CAIR minus CpG/ αCTLA4	CAIR minus αCD40/ αCTLA4	CAIR minus CpG/ αCD40
RT	**<0.0001**	**<0.0001**	**<0.0001**	**<0.0001**	**<0.0001**	0.0469	**<0.0001**	**0.0007**	**0.0001**
CAIR		0.6439	0.877	0.9903	**<0.0001**	**<0.0001**	>0.9999	**<0.0001**	**<0.0001**
CAIR minus CpG			>0.9999	0.9963	**<0.0001**	**<0.0001**	0.7636	**<0.0001**	**<0.0001**
CAIR minus αCD40				>0.9999	**<0.0001**	**<0.0001**	0.9221	**<0.0001**	**<0.0001**
CAIR minus αCTLA4					**<0.0001**	**<0.0001**	0.9936	**<0.0001**	**<0.0001**
CAIR minus RT						0.2848	**<0.0001**	0.9444	>0.9999
CAIR minus IC							**<0.0001**	0.9785	0.4559
CAIR minus CpG/αCTLA4								**<0.0001**	**<0.0001**
CAIR minus αCD40/αCTLA4									0.9593

<u>**Supplementary Table S3:** Statistical output of comparisons of response rate for each treatment group represented in Figures 2 and 3.</u>

Treatment	CAIR	CAIR minus CpG	CAIR minus αCD40	CAIR minus αCTLA4	CAIR minus RT	CAIR minus IC	CAIR minus CpG/αCTLA4	CAIR minus αCD40/αCTLA4	CAIR minus CpG/αCD40
RT	***	***	***	**	ns	ns	ns	ns	ns
CAIR		ns	ns	ns	*	**	ns	*	ns
CAIR minus CpG			ns	ns	*	**	ns	*	ns
CAIR minus αCD40				ns	*	**	ns	*	ns
CAIR minus αCTLA4					*	**	ns	*	ns
CAIR minus RT						ns	ns	ns	ns
CAIR minus IC							ns	ns	ns
CAIR minus CpG/αCTLA4								ns	ns
CAIR minus αCD40/αCTLA4									ns

ns = not significant; *p<0.05; **p<0.01; ***p<0.001

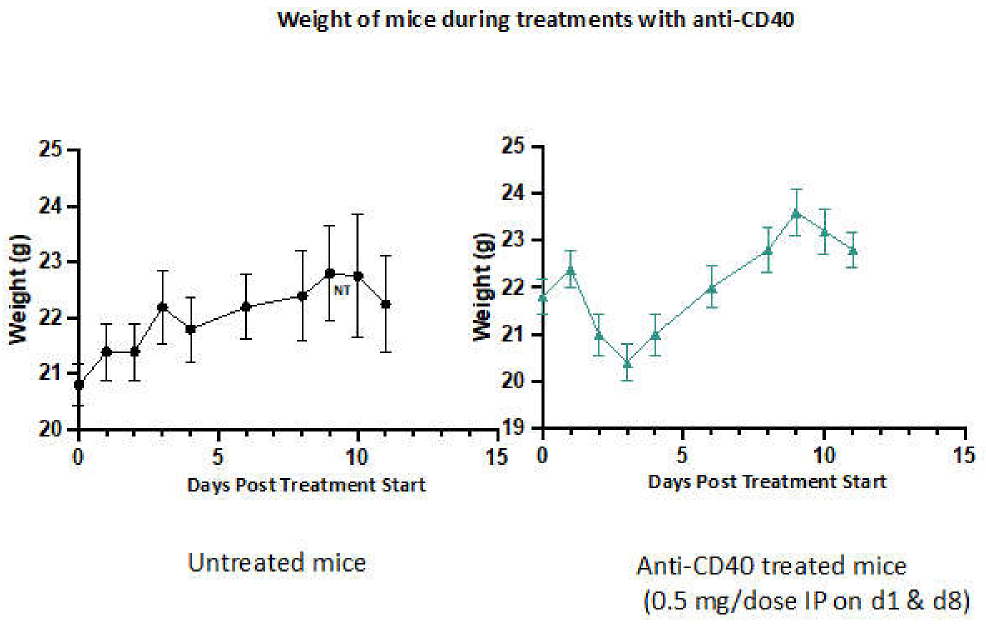

Supplemental Figure S1: C56BL/6 mice bearing 17 mm^3 9464D-GD2 tumors were not treated, or received anti-CD40 (0.5 mg/mouse intraperitoneally) on days 1 and 8. Mouse weights were obtained every 2–3 days. There were 5 mice per group. Mouse weight dropped ~10% over 4 days following the first dose, with minimal weight loss after the 2nd dose.
